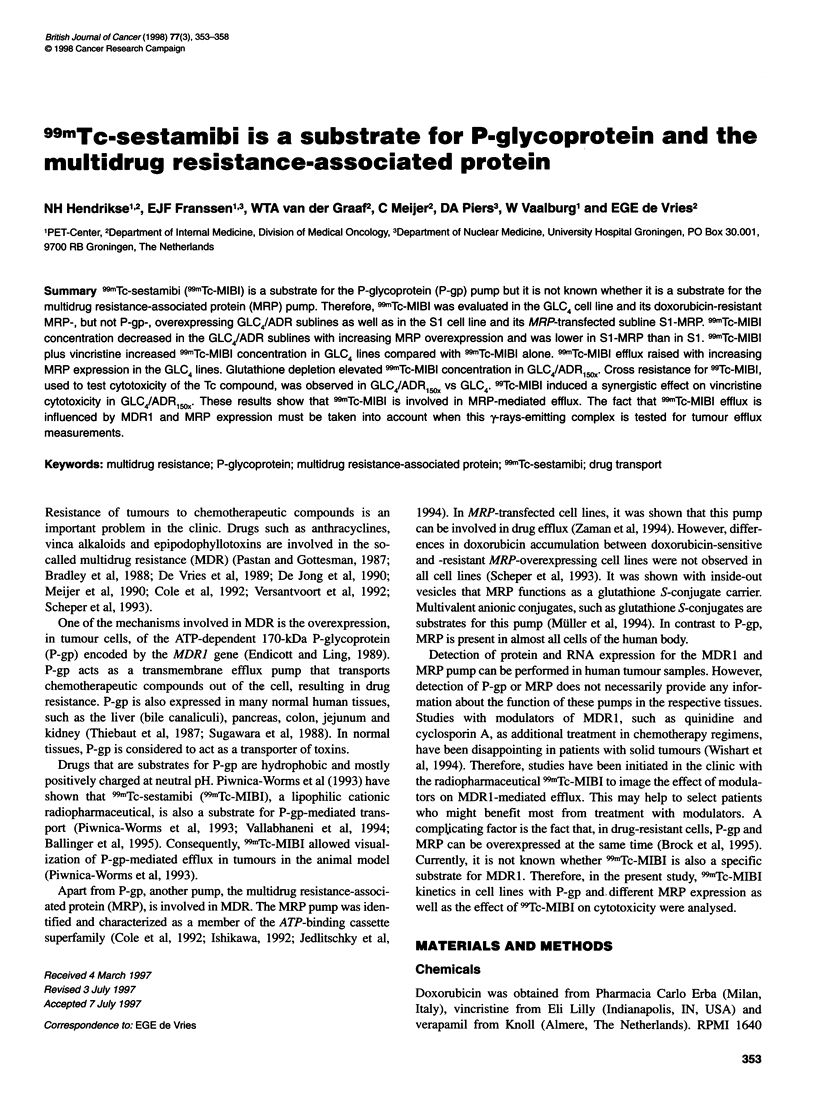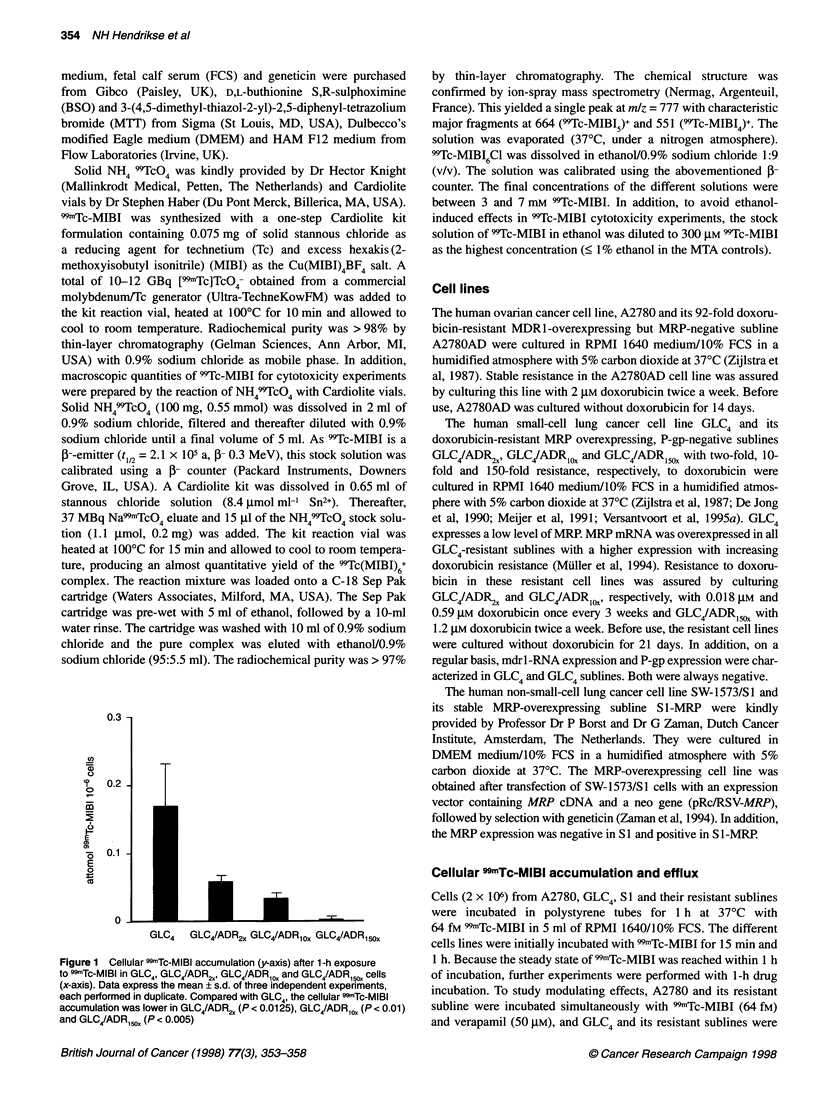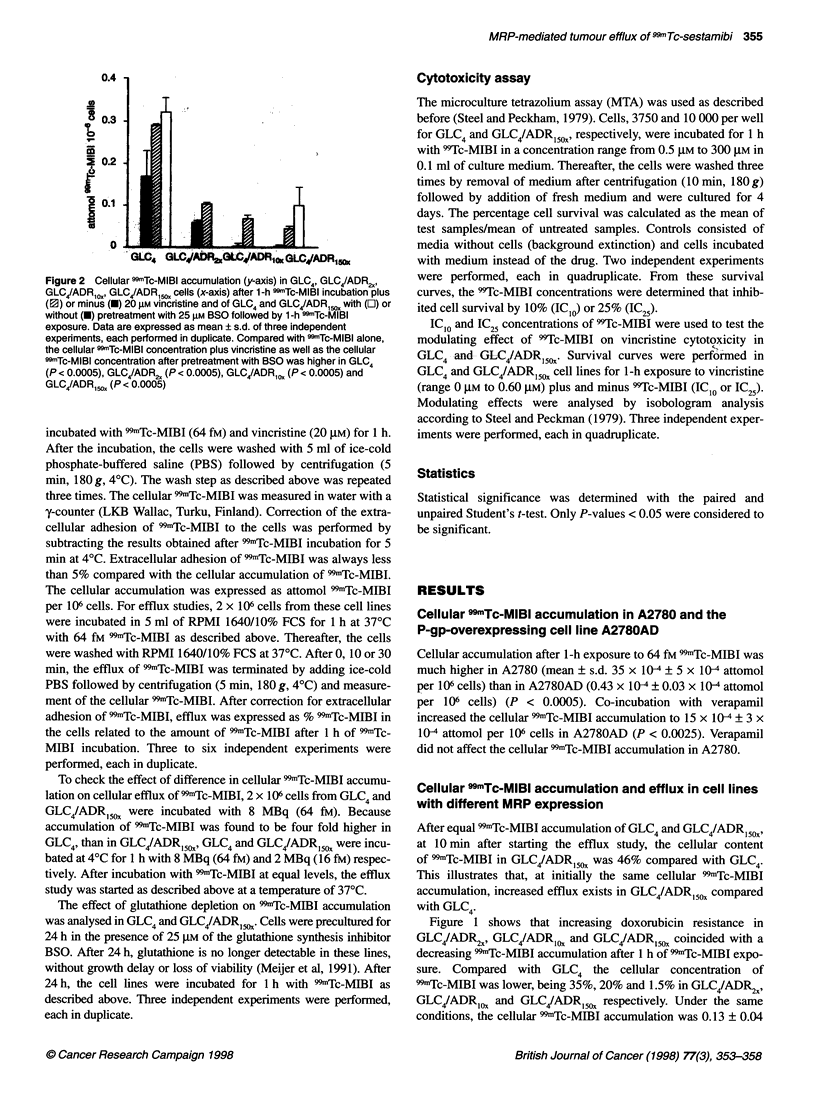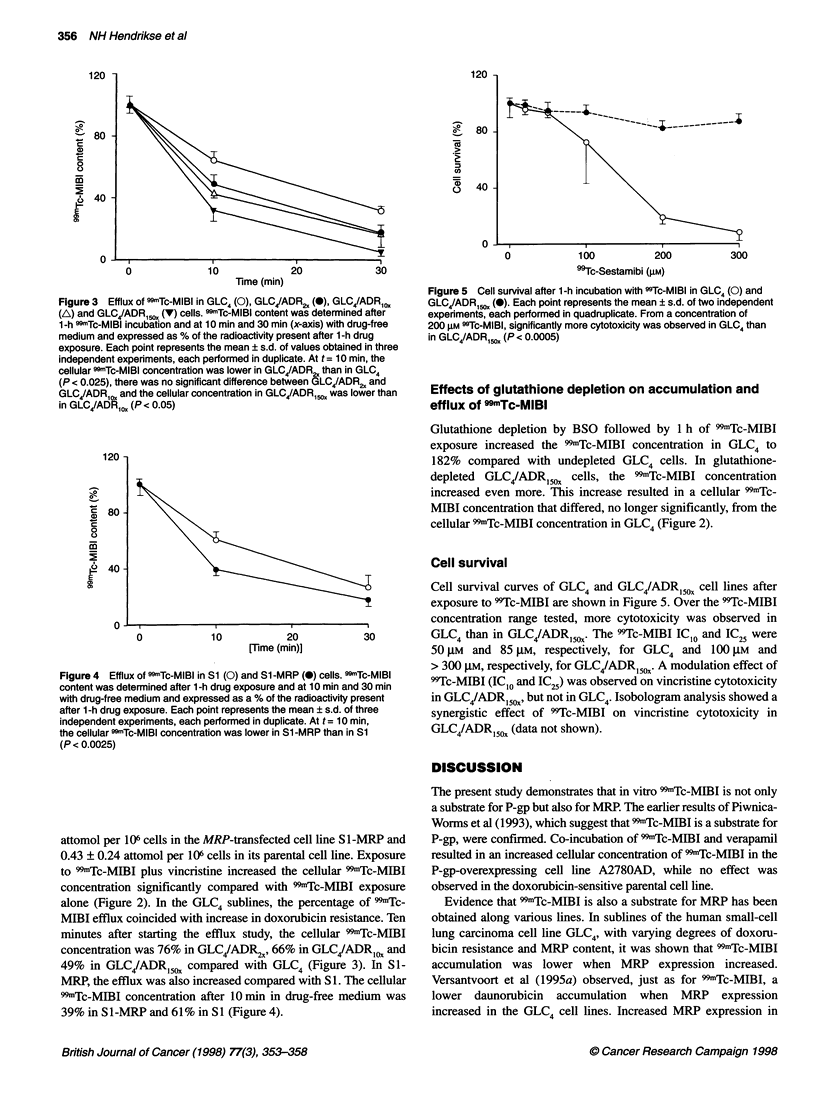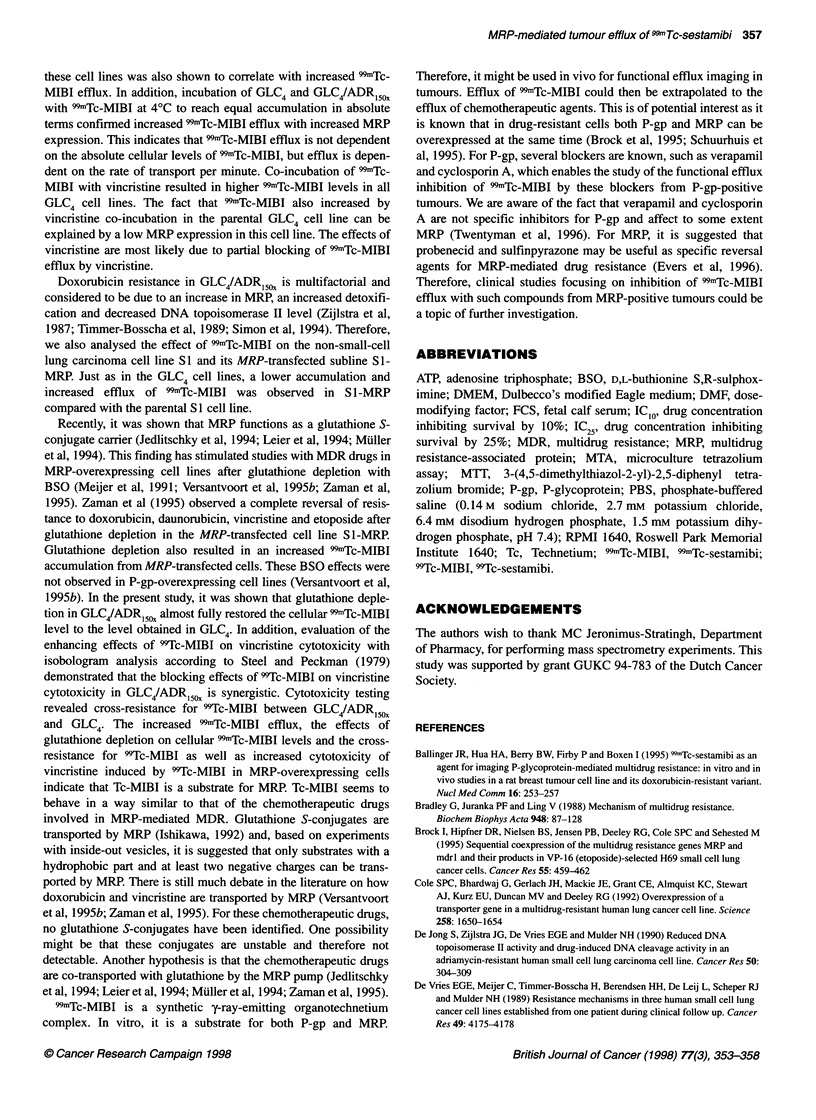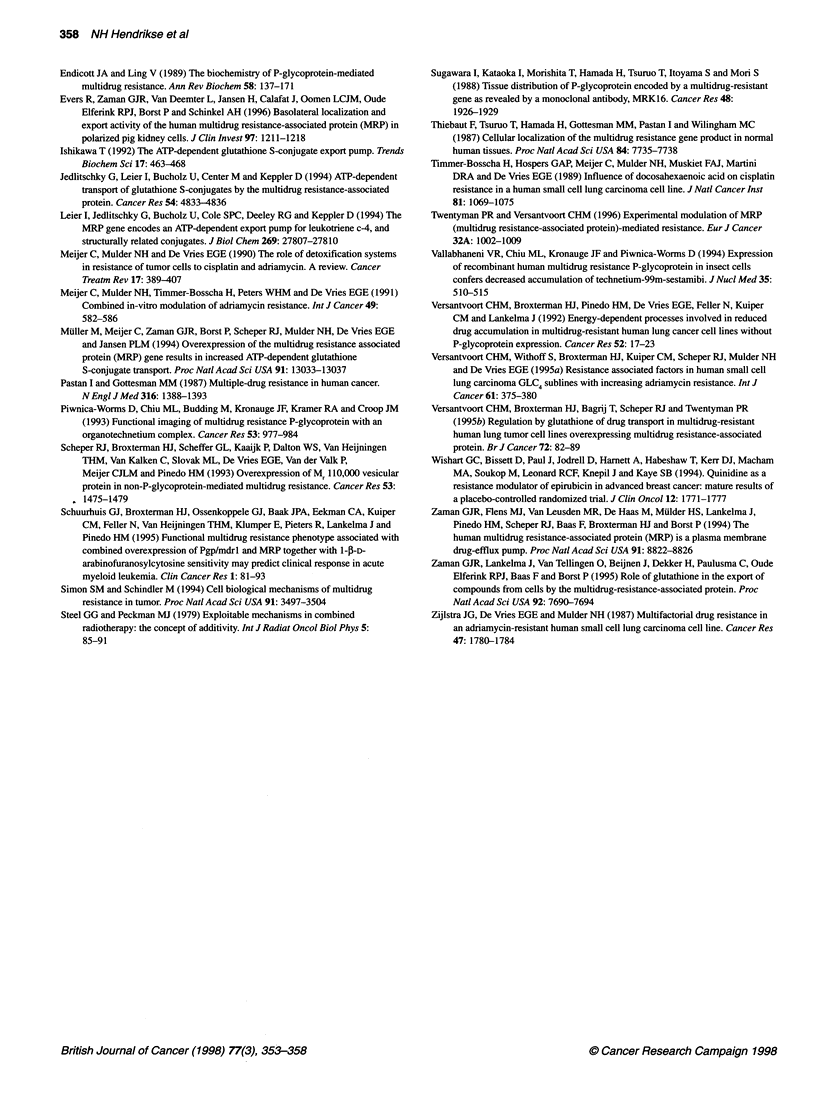# 99mTc-sestamibi is a substrate for P-glycoprotein and the multidrug resistance-associated protein.

**DOI:** 10.1038/bjc.1998.57

**Published:** 1998

**Authors:** N. H. Hendrikse, E. J. Franssen, W. T. van der Graaf, C. Meijer, D. A. Piers, W. Vaalburg, E. G. de Vries

**Affiliations:** PET-Center, Department of Internal Medicine, University Hospital Groningen, The Netherlands.

## Abstract

99mTc-sestamibi (99mTc-MIBI) is a substrate for the P-glycoprotein (P-gp) pump but it is not known whether it is a substrate for the multidrug resistance-associated protein (MRP) pump. Therefore, 99mTc-MIBI was evaluated in the GLC4 cell line and its doxorubicin-resistant MRP-, but not P-gp-, overexpressing GLC4/ADR sublines as well as in the S1 cell line and its MRP-transfected subline S1-MRP. 99mTc-MIBI concentration decreased in the GLC4/ADR sublines with increasing MRP overexpression and was lower in S1-MRP than in S1. 99mTc-MIBI plus vincristine increased 99mTc-MIBI concentration in GLC4 lines compared with 99mTc-MIBI alone. 99mTc-MIBI efflux raised with increasing MRP expression in the GLC4 lines. Glutathione depletion elevated 99mTc-MIBI concentration in GLC4/ADR150x. Cross resistance for 99Tc-MIBI, used to test cytotoxicity of the Tc compound, was observed in GLC4/ADR150x vs GLC4. 99Tc-MIBI induced a synergistic effect on vincristine cytotoxicity in GLC4/ADR150x. These results show that 99mTc-MIBI is involved in MRP-mediated efflux. The fact that 99mTc-MIBI efflux is influenced by MDR1 and MRP expression must be taken into account when this gamma-rays-emitting complex is tested for tumour efflux measurements.


British Journal of Cancer (1998) 77(3), 353–358
© 1998 Cancer Research Campaign

### $^{99m}$Tc-sestamibi is a substrate for P-glycoprotein and the multidrug resistance-associated protein

NH Hendrikse[1,2], EJF Franssen[1,3], WTA van der Graaf[2], C Meijer[2], DA Piers[3], W Vaalburg[1] and EGE de Vries[2]

[1]PET-Center, [2]Department of Internal Medicine, Division of Medical Oncology, [3]Department of Nuclear Medicine, University Hospital Groningen, PO Box 30.001, 9700 RB Groningen, The Netherlands

**Summary** $^{99m}$Tc-sestamibi ($^{99m}$Tc-MIBI) is a substrate for the P-glycoprotein (P-gp) pump but it is not known whether it is a substrate for the multidrug resistance-associated protein (MRP) pump. Therefore, $^{99m}$Tc-MIBI was evaluated in the $GLC_4$ cell line and its doxorubicin-resistant MRP-, but not P-gp-, overexpressing $GLC_4$/ADR sublines as well as in the S1 cell line and its *MRP*-transfected subline S1-MRP. $^{99m}$Tc-MIBI concentration decreased in the $GLC_4$/ADR sublines with increasing MRP overexpression and was lower in S1-MRP than in S1. $^{99m}$Tc-MIBI plus vincristine increased $^{99m}$Tc-MIBI concentration in $GLC_4$ lines compared with $^{99m}$Tc-MIBI alone. $^{99m}$Tc-MIBI efflux raised with increasing MRP expression in the $GLC_4$ lines. Glutathione depletion elevated $^{99m}$Tc-MIBI concentration in $GLC_4/ADR_{150x}$. Cross resistance for $^{99}$Tc-MIBI, used to test cytotoxicity of the Tc compound, was observed in $GLC_4/ADR_{150x}$ vs $GLC_4$. $^{99}$Tc-MIBI induced a synergistic effect on vincristine cytotoxicity in $GLC_4/ADR_{150x}$. These results show that $^{99m}$Tc-MIBI is involved in MRP-mediated efflux. The fact that $^{99m}$Tc-MIBI efflux is influenced by MDR1 and MRP expression must be taken into account when this $\gamma$-rays-emitting complex is tested for tumour efflux measurements.

**Keywords:** multidrug resistance; P-glycoprotein; multidrug resistance-associated protein; $^{99m}$Tc-sestamibi; drug transport

Resistance of tumours to chemotherapeutic compounds is an important problem in the clinic. Drugs such as anthracyclines, vinca alkaloids and epipodophyllotoxins are involved in the so-called multidrug resistance (MDR) (Pastan and Gottesman, 1987; Bradley et al, 1988; De Vries et al, 1989; De Jong et al, 1990; Meijer et al, 1990; Cole et al, 1992; Versantvoort et al, 1992; Scheper et al, 1993).

One of the mechanisms involved in MDR is the overexpression, in tumour cells, of the ATP-dependent 170-kDa P-glycoprotein (P-gp) encoded by the *MDR1* gene (Endicott and Ling, 1989). P-gp acts as a transmembrane efflux pump that transports chemotherapeutic compounds out of the cell, resulting in drug resistance. P-gp is also expressed in many normal human tissues, such as the liver (bile canaliculi), pancreas, colon, jejunum and kidney (Thiebaut et al, 1987; Sugawara et al, 1988). In normal tissues, P-gp is considered to act as a transporter of toxins.

Drugs that are substrates for P-gp are hydrophobic and mostly positively charged at neutral pH. Piwnica-Worms et al (1993) have shown that $^{99m}$Tc-sestamibi ($^{99m}$Tc-MIBI), a lipophilic cationic radiopharmaceutical, is also a substrate for P-gp-mediated transport (Piwnica-Worms et al, 1993; Vallabhaneni et al, 1994; Ballinger et al, 1995). Consequently, $^{99m}$Tc-MIBI allowed visualization of P-gp-mediated efflux in tumours in the animal model (Piwnica-Worms et al, 1993).

Apart from P-gp, another pump, the multidrug resistance-associated protein (MRP), is involved in MDR. The MRP pump was identified and characterized as a member of the *ATP*-binding cassette superfamily (Cole et al, 1992; Ishikawa, 1992; Jedlitschky et al, 1994). In *MRP*-transfected cell lines, it was shown that this pump can be involved in drug efflux (Zaman et al, 1994). However, differences in doxorubicin accumulation between doxorubicin-sensitive and -resistant *MRP*-overexpressing cell lines were not observed in all cell lines (Scheper et al, 1993). It was shown with inside-out vesicles that MRP functions as a glutathione *S*-conjugate carrier. Multivalent anionic conjugates, such as glutathione *S*-conjugates are substrates for this pump (Müller et al, 1994). In contrast to P-gp, MRP is present in almost all cells of the human body.

Detection of protein and RNA expression for the MDR1 and MRP pump can be performed in human tumour samples. However, detection of P-gp or MRP does not necessarily provide any information about the function of these pumps in the respective tissues. Studies with modulators of MDR1, such as quinidine and cyclosporin A, as additional treatment in chemotherapy regimens, have been disappointing in patients with solid tumours (Wishart et al, 1994). Therefore, studies have been initiated in the clinic with the radiopharmaceutical $^{99m}$Tc-MIBI to image the effect of modulators on MDR1-mediated efflux. This may help to select patients who might benefit most from treatment with modulators. A complicating factor is the fact that, in drug-resistant cells, P-gp and MRP can be overexpressed at the same time (Brock et al, 1995). Currently, it is not known whether $^{99m}$Tc-MIBI is also a specific substrate for MDR1. Therefore, in the present study, $^{99m}$Tc-MIBI kinetics in cell lines with P-gp and different MRP expression as well as the effect of $^{99}$Tc-MIBI on cytotoxicity were analysed.

*Received 4 March 1997*
*Revised 3 July 1997*
*Accepted 7 July 1997*

*Correspondence to:* EGE de Vries

### MATERIALS AND METHODS

### Chemicals

Doxorubicin was obtained from Pharmacia Carlo Erba (Milan, Italy), vincristine from Eli Lilly (Indianapolis, IN, USA) and verapamil from Knoll (Almere, The Netherlands). RPMI 1640

medium, fetal calf serum (FCS) and geneticin were purchased from Gibco (Paisley, UK), D,L-buthionine S,R-sulphoximine (BSO) and 3-(4,5-dimethyl-thiazol-2-yl)-2,5-diphenyl-tetrazolium bromide (MTT) from Sigma (St Louis, MD, USA), Dulbecco's modified Eagle medium (DMEM) and HAM F12 medium from Flow Laboratories (Irvine, UK).

Solid $NH_4$ $^{99}TcO_4$ was kindly provided by Dr Hector Knight (Mallinkrodt Medical, Petten, The Netherlands) and Cardiolite vials by Dr Stephen Haber (Du Pont Merck, Billerica, MA, USA). $^{99m}$Tc-MIBI was synthesized with a one-step Cardiolite kit formulation containing 0.075 mg of solid stannous chloride as a reducing agent for technetium (Tc) and excess hexakis(2-methoxyisobutyl isonitrile) (MIBI) as the $Cu(MIBI)_4BF_4$ salt. A total of 10–12 GBq $[^{99m}Tc]TcO_4^-$ obtained from a commercial molybdenum/Tc generator (Ultra-TechneKowFM) was added to the kit reaction vial, heated at 100°C for 10 min and allowed to cool to room temperature. Radiochemical purity was > 98% by thin-layer chromatography (Gelman Sciences, Ann Arbor, MI, USA) with 0.9% sodium chloride as mobile phase. In addition, macroscopic quantities of $^{99}$Tc-MIBI for cytotoxicity experiments were prepared by the reaction of $NH_4{}^{99}TcO_4$ with Cardiolite vials. Solid $NH_4{}^{99}TcO_4$ (100 mg, 0.55 mmol) was dissolved in 2 ml of 0.9% sodium chloride, filtered and thereafter diluted with 0.9% sodium chloride until a final volume of 5 ml. As $^{99}$Tc-MIBI is a $\beta^-$-emitter ($t_{1/2} = 2.1 \times 10^5$ a, $\beta^-$ 0.3 MeV), this stock solution was calibrated using a $\beta^-$ counter (Packard Instruments, Downers Grove, IL, USA). A Cardiolite kit was dissolved in 0.65 ml of stannous chloride solution (8.4 µmol $ml^{-1}$ $Sn^{2+}$). Thereafter, 37 MBq $Na^{99m}TcO_4$ eluate and 15 µl of the $NH_4{}^{99}TcO_4$ stock solution (1.1 µmol, 0.2 mg) was added. The kit reaction vial was heated at 100°C for 15 min and allowed to cool to room temperature, producing an almost quantitative yield of the $^{99}Tc(MIBI)_6^+$ complex. The reaction mixture was loaded onto a C-18 Sep Pak cartridge (Waters Associates, Milford, MA, USA). The Sep Pak cartridge was pre-wet with 5 ml of ethanol, followed by a 10-ml water rinse. The cartridge was washed with 10 ml of 0.9% sodium chloride and the pure complex was eluted with ethanol/0.9% sodium chloride (95:5.5 ml). The radiochemical purity was > 97% by thin-layer chromatography. The chemical structure was confirmed by ion-spray mass spectrometry (Nermag, Argenteuil, France). This yielded a single peak at $m/z = 777$ with characteristic major fragments at 664 $(^{99}Tc\text{-}MIBI_5)^+$ and 551 $(^{99}Tc\text{-}MIBI_4)^+$. The solution was evaporated (37°C, under a nitrogen atmosphere). $^{99}Tc\text{-}MIBI_6Cl$ was dissolved in ethanol/0.9% sodium chloride 1:9 (v/v). The solution was calibrated using the abovementioned $\beta^-$ counter. The final concentrations of the different solutions were between 3 and 7 mM $^{99}$Tc-MIBI. In addition, to avoid ethanol-induced effects in $^{99}$Tc-MIBI cytotoxicity experiments, the stock solution of $^{99}$Tc-MIBI in ethanol was diluted to 300 µM $^{99}$Tc-MIBI as the highest concentration (≤ 1% ethanol in the MTA controls).

**Figure 1** Cellular $^{99m}$Tc-MIBI accumulation (*y*-axis) after 1-h exposure to $^{99m}$Tc-MIBI in $GLC_4$, $GLC_4/ADR_{2x}$, $GLC_4/ADR_{10x}$ and $GLC_4/ADR_{150x}$ cells (*x*-axis). Data express the mean ± s.d. of three independent experiments, each performed in duplicate. Compared with $GLC_4$, the cellular $^{99m}$Tc-MIBI accumulation was lower in $GLC_4/ADR_{2x}$ ($P < 0.0125$), $GLC_4/ADR_{10x}$ ($P < 0.01$) and $GLC_4/ADR_{150x}$ ($P < 0.005$)

### Cell lines

The human ovarian cancer cell line, A2780 and its 92-fold doxorubicin-resistant MDR1-overexpressing but MRP-negative subline A2780AD were cultured in RPMI 1640 medium/10% FCS in a humidified atmosphere with 5% carbon dioxide at 37°C (Zijlstra et al, 1987). Stable resistance in the A2780AD cell line was assured by culturing this line with 2 µM doxorubicin twice a week. Before use, A2780AD was cultured without doxorubicin for 14 days.

The human small-cell lung cancer cell line $GLC_4$ and its doxorubicin-resistant MRP overexpressing, P-gp-negative sublines $GLC_4/ADR_{2x}$, $GLC_4/ADR_{10x}$ and $GLC_4/ADR_{150x}$ with two-fold, 10-fold and 150-fold resistance, respectively, to doxorubicin were cultured in RPMI 1640 medium/10% FCS in a humidified atmosphere with 5% carbon dioxide at 37°C (Zijlstra et al, 1987; De Jong et al, 1990; Meijer et al, 1991; Versantvoort et al, 1995*a*). $GLC_4$ expresses a low level of MRP. MRP mRNA was overexpressed in all $GLC_4$-resistant sublines with a higher expression with increasing doxorubicin resistance (Müller et al, 1994). Resistance to doxorubicin in these resistant cell lines was assured by culturing $GLC_4/ADR_{2x}$ and $GLC_4/ADR_{10x}$, respectively, with 0.018 µM and 0.59 µM doxorubicin once every 3 weeks and $GLC_4/ADR_{150x}$ with 1.2 µM doxorubicin twice a week. Before use, the resistant cell lines were cultured without doxorubicin for 21 days. In addition, on a regular basis, mdr1-RNA expression and P-gp expression were characterized in $GLC_4$ and $GLC_4$ sublines. Both were always negative.

The human non-small-cell lung cancer cell line SW-1573/S1 and its stable MRP-overexpressing subline S1-MRP were kindly provided by Professor Dr P Borst and Dr G Zaman, Dutch Cancer Institute, Amsterdam, The Netherlands. They were cultured in DMEM medium/10% FCS in a humidified atmosphere with 5% carbon dioxide at 37°C. The MRP-overexpressing cell line was obtained after transfection of SW-1573/S1 cells with an expression vector containing *MRP* cDNA and a neo gene (pRc/RSV-*MRP*), followed by selection with geneticin (Zaman et al, 1994). In addition, the MRP expression was negative in S1 and positive in S1-MRP.

### Cellular $^{99m}$Tc-MIBI accumulation and efflux

Cells ($2 \times 10^6$) from A2780, $GLC_4$, S1 and their resistant sublines were incubated in polystyrene tubes for 1 h at 37°C with 64 fM $^{99m}$Tc-MIBI in 5 ml of RPMI 1640/10% FCS. The different cells lines were initially incubated with $^{99m}$Tc-MIBI for 15 min and 1 h. Because the steady state of $^{99m}$Tc-MIBI was reached within 1 h of incubation, further experiments were performed with 1-h drug incubation. To study modulating effects, A2780 and its resistant subline were incubated simultaneously with $^{99m}$Tc-MIBI (64 fM) and verapamil (50 µM), and $GLC_4$ and its resistant sublines were

© Cancer Research Campaign 1998

**Figure 2** Cellular $^{99m}$Tc-MIBI accumulation (*y*-axis) in $GLC_4$, $GLC_4/ADR_{2x}$, $GLC_4/ADR_{10x}$, $GLC_4/ADR_{150x}$ cells (*x*-axis) after 1-h $^{99m}$Tc-MIBI incubation plus (▨) or minus (■) 20 µM vincristine and of $GLC_4$ and $GLC_4/ADR_{150x}$ with (□) or without (■) pretreatment with 25 µM BSO followed by 1-h $^{99m}$Tc-MIBI exposure. Data are expressed as mean ± s.d. of three independent experiments, each performed in duplicate. Compared with $^{99m}$Tc-MIBI alone, the cellular $^{99m}$Tc-MIBI concentration plus vincristine as well as the cellular $^{99m}$Tc-MIBI concentration after pretreatment with BSO was higher in $GLC_4$ ($P < 0.0005$), $GLC_4/ADR_{2x}$ ($P < 0.0005$), $GLC_4/ADR_{10x}$ ($P < 0.0005$) and $GLC_4/ADR_{150x}$ ($P < 0.0005$)

incubated with $^{99m}$Tc-MIBI (64 fM) and vincristine (20 µM) for 1 h. After the incubation, the cells were washed with 5 ml of ice-cold phosphate-buffered saline (PBS) followed by centrifugation (5 min, 180 *g*, 4°C). The wash step as described above was repeated three times. The cellular $^{99m}$Tc-MIBI was measured in water with a γ-counter (LKB Wallac, Turku, Finland). Correction of the extracellular adhesion of $^{99m}$Tc-MIBI to the cells was performed by subtracting the results obtained after $^{99m}$Tc-MIBI incubation for 5 min at 4°C. Extracellular adhesion of $^{99m}$Tc-MIBI was always less than 5% compared with the cellular accumulation of $^{99m}$Tc-MIBI. The cellular accumulation was expressed as attomol $^{99m}$Tc-MIBI per $10^6$ cells. For efflux studies, $2 \times 10^6$ cells from these cell lines were incubated in 5 ml of RPMI 1640/10% FCS for 1 h at 37°C with 64 fM $^{99m}$Tc-MIBI as described above. Thereafter, the cells were washed with RPMI 1640/10% FCS at 37°C. After 0, 10 or 30 min, the efflux of $^{99m}$Tc-MIBI was terminated by adding ice-cold PBS followed by centrifugation (5 min, 180 *g*, 4°C) and measurement of the cellular $^{99m}$Tc-MIBI. After correction for extracellular adhesion of $^{99m}$Tc-MIBI, efflux was expressed as % $^{99m}$Tc-MIBI in the cells related to the amount of $^{99m}$Tc-MIBI after 1 h of $^{99m}$Tc-MIBI incubation. Three to six independent experiments were performed, each in duplicate.

To check the effect of difference in cellular $^{99m}$Tc-MIBI accumulation on cellular efflux of $^{99m}$Tc-MIBI, $2 \times 10^6$ cells from $GLC_4$ and $GLC_4/ADR_{150x}$ were incubated with 8 MBq (64 fM). Because accumulation of $^{99m}$Tc-MIBI was found to be four fold higher in $GLC_4$, than in $GLC_4/ADR_{150x}$, $GLC_4$ and $GLC_4/ADR_{150x}$ were incubated at 4°C for 1 h with 8 MBq (64 fM) and 2 MBq (16 fM) respectively. After incubation with $^{99m}$Tc-MIBI at equal levels, the efflux study was started as described above at a temperature of 37°C.

The effect of glutathione depletion on $^{99m}$Tc-MIBI accumulation was analysed in $GLC_4$ and $GLC_4/ADR_{150x}$. Cells were precultured for 24 h in the presence of 25 µM of the glutathione synthesis inhibitor BSO. After 24 h, glutathione is no longer detectable in these lines, without growth delay or loss of viability (Meijer et al, 1991). After 24 h, the cell lines were incubated for 1 h with $^{99m}$Tc-MIBI as described above. Three independent experiments were performed, each in duplicate.

### Cytotoxicity assay

The microculture tetrazolium assay (MTA) was used as described before (Steel and Peckham, 1979). Cells, 3750 and 10 000 per well for $GLC_4$ and $GLC_4/ADR_{150x}$, respectively, were incubated for 1 h with $^{99}$Tc-MIBI in a concentration range from 0.5 µM to 300 µM in 0.1 ml of culture medium. Thereafter, the cells were washed three times by removal of medium after centrifugation (10 min, 180 *g*) followed by addition of fresh medium and were cultured for 4 days. The percentage cell survival was calculated as the mean of test samples/mean of untreated samples. Controls consisted of media without cells (background extinction) and cells incubated with medium instead of the drug. Two independent experiments were performed, each in quadruplicate. From these survival curves, the $^{99}$Tc-MIBI concentrations were determined that inhibited cell survival by 10% ($IC_{10}$) or 25% ($IC_{25}$).

$IC_{10}$ and $IC_{25}$ concentrations of $^{99}$Tc-MIBI were used to test the modulating effect of $^{99}$Tc-MIBI on vincristine cytotoxicity in $GLC_4$ and $GLC_4/ADR_{150x}$. Survival curves were performed in $GLC_4$ and $GLC_4/ADR_{150x}$ cell lines for 1-h exposure to vincristine (range 0 µM to 0.60 µM) plus and minus $^{99}$Tc-MIBI ($IC_{10}$ or $IC_{25}$). Modulating effects were analysed by isobologram analysis according to Steel and Peckman (1979). Three independent experiments were performed, each in quadruplicate.

### Statistics

Statistical significance was determined with the paired and unpaired Student's *t*-test. Only *P*-values < 0.05 were considered to be significant.

### RESULTS

### Cellular $^{99m}$Tc-MIBI accumulation in A2780 and the P-gp-overexpressing cell line A2780AD

Cellular accumulation after 1-h exposure to 64 fM $^{99m}$Tc-MIBI was much higher in A2780 (mean ± s.d. $35 \times 10^{-4} \pm 5 \times 10^{-4}$ attomol per $10^6$ cells) than in A2780AD ($0.43 \times 10^{-4} \pm 0.03 \times 10^{-4}$ attomol per $10^6$ cells) ($P < 0.0005$). Co-incubation with verapamil increased the cellular $^{99m}$Tc-MIBI accumulation to $15 \times 10^{-4} \pm 3 \times 10^{-4}$ attomol per $10^6$ cells in A2780AD ($P < 0.0025$). Verapamil did not affect the cellular $^{99m}$Tc-MIBI accumulation in A2780.

### Cellular $^{99m}$Tc-MIBI accumulation and efflux in cell lines with different MRP expression

After equal $^{99m}$Tc-MIBI accumulation of $GLC_4$ and $GLC_4/ADR_{150x}$, at 10 min after starting the efflux study, the cellular content of $^{99m}$Tc-MIBI in $GLC_4/ADR_{150x}$ was 46% compared with $GLC_4$. This illustrates that, at initially the same cellular $^{99m}$Tc-MIBI accumulation, increased efflux exists in $GLC_4/ADR_{150x}$ compared with $GLC_4$.

Figure 1 shows that increasing doxorubicin resistance in $GLC_4/ADR_{2x}$, $GLC_4/ADR_{10x}$ and $GLC_4/ADR_{150x}$ coincided with a decreasing $^{99m}$Tc-MIBI accumulation after 1 h of $^{99m}$Tc-MIBI exposure. Compared with $GLC_4$ the cellular concentration of $^{99m}$Tc-MIBI was lower, being 35%, 20% and 1.5% in $GLC_4/ADR_{2x}$, $GLC_4/ADR_{10x}$ and $GLC_4/ADR_{150x}$ respectively. Under the same conditions, the cellular $^{99m}$Tc-MIBI accumulation was 0.13 ± 0.04

© Cancer Research Campaign 1998

Figure 3 Efflux of $^{99m}$Tc-MIBI in $GLC_4$ (○), $GLC_4/ADR_{2x}$ (●), $GLC_4/ADR_{10x}$ (△) and $GLC_4/ADR_{150x}$ (▼) cells. $^{99m}$Tc-MIBI content was determined after 1-h $^{99m}$Tc-MIBI incubation and at 10 min and 30 min (*x*-axis) with drug-free medium and expressed as % of the radioactivity present after 1-h drug exposure. Each point represents the mean ± s.d. of values obtained in three independent experiments, each performed in duplicate. At $t = 10$ min, the cellular $^{99m}$Tc-MIBI concentration was lower in $GLC_4/ADR_{2x}$ than in $GLC_4$ ($P < 0.025$), there was no significant difference between $GLC_4/ADR_{2x}$ and $GLC_4/ADR_{10x}$ and the cellular concentration in $GLC_4/ADR_{150x}$ was lower than in $GLC_4/ADR_{10x}$ ($P < 0.05$)

Figure 4 Efflux of $^{99m}$Tc-MIBI in S1 (○) and S1-MRP (●) cells. $^{99m}$Tc-MIBI content was determined after 1-h drug exposure and at 10 min and 30 min with drug-free medium and expressed as a % of the radioactivity present after 1-h drug exposure. Each point represents the mean ± s.d. of three independent experiments, each performed in duplicate. At $t = 10$ min, the cellular $^{99m}$Tc-MIBI concentration was lower in S1-MRP than in S1 ($P < 0.0025$)

attomol per $10^6$ cells in the *MRP*-transfected cell line S1-MRP and 0.43 ± 0.24 attomol per $10^6$ cells in its parental cell line. Exposure to $^{99m}$Tc-MIBI plus vincristine increased the cellular $^{99m}$Tc-MIBI concentration significantly compared with $^{99m}$Tc-MIBI exposure alone (Figure 2). In the $GLC_4$ sublines, the percentage of $^{99m}$Tc-MIBI efflux coincided with increase in doxorubicin resistance. Ten minutes after starting the efflux study, the cellular $^{99m}$Tc-MIBI concentration was 76% in $GLC_4/ADR_{2x}$, 66% in $GLC_4/ADR_{10x}$ and 49% in $GLC_4/ADR_{150x}$ compared with $GLC_4$ (Figure 3). In S1-MRP, the efflux was also increased compared with S1. The cellular $^{99m}$Tc-MIBI concentration after 10 min in drug-free medium was 39% in S1-MRP and 61% in S1 (Figure 4).

Figure 5 Cell survival after 1-h incubation with $^{99}$Tc-MIBI in $GLC_4$ (○) and $GLC_4/ADR_{150x}$ (●). Each point represents the mean ± s.d. of two independent experiments, each performed in quadruplicate. From a concentration of 200 μM $^{99}$Tc-MIBI, significantly more cytotoxicity was observed in $GLC_4$ than in $GLC_4/ADR_{150x}$ ($P < 0.0005$)

### Effects of glutathione depletion on accumulation and efflux of $^{99m}$Tc-MIBI

Glutathione depletion by BSO followed by 1 h of $^{99m}$Tc-MIBI exposure increased the $^{99m}$Tc-MIBI concentration in $GLC_4$ to 182% compared with undepleted $GLC_4$ cells. In glutathione-depleted $GLC_4/ADR_{150x}$ cells, the $^{99m}$Tc-MIBI concentration increased even more. This increase resulted in a cellular $^{99m}$Tc-MIBI concentration that differed, no longer significantly, from the cellular $^{99m}$Tc-MIBI concentration in $GLC_4$ (Figure 2).

### Cell survival

Cell survival curves of $GLC_4$ and $GLC_4/ADR_{150x}$ cell lines after exposure to $^{99}$Tc-MIBI are shown in Figure 5. Over the $^{99}$Tc-MIBI concentration range tested, more cytotoxicity was observed in $GLC_4$ than in $GLC_4/ADR_{150x}$. The $^{99}$Tc-MIBI $IC_{10}$ and $IC_{25}$ were 50 μM and 85 μM, respectively, for $GLC_4$ and 100 μM and > 300 μM, respectively, for $GLC_4/ADR_{150x}$. A modulation effect of $^{99}$Tc-MIBI ($IC_{10}$ and $IC_{25}$) was observed on vincristine cytotoxicity in $GLC_4/ADR_{150x}$, but not in $GLC_4$. Isobologram analysis showed a synergistic effect of $^{99}$Tc-MIBI on vincristine cytotoxicity in $GLC_4/ADR_{150x}$ (data not shown).

### DISCUSSION

The present study demonstrates that in vitro $^{99m}$Tc-MIBI is not only a substrate for P-gp but also for MRP. The earlier results of Piwnica-Worms et al (1993), which suggest that $^{99m}$Tc-MIBI is a substrate for P-gp, were confirmed. Co-incubation of $^{99m}$Tc-MIBI and verapamil resulted in an increased cellular concentration of $^{99m}$Tc-MIBI in the P-gp-overexpressing cell line A2780AD, while no effect was observed in the doxorubicin-sensitive parental cell line.

Evidence that $^{99m}$Tc-MIBI is also a substrate for MRP has been obtained along various lines. In sublines of the human small-cell lung carcinoma cell line $GLC_4$, with varying degrees of doxorubicin resistance and MRP content, it was shown that $^{99m}$Tc-MIBI accumulation was lower when MRP expression increased. Versantvoort et al (1995*a*) observed, just as for $^{99m}$Tc-MIBI, a lower daunorubicin accumulation when MRP expression increased in the $GLC_4$ cell lines. Increased MRP expression in

© Cancer Research Campaign 1998

these cell lines was also shown to correlate with increased $^{99m}$Tc-MIBI efflux. In addition, incubation of $GLC_4$ and $GLC_4/ADR_{150x}$ with $^{99m}$Tc-MIBI at 4°C to reach equal accumulation in absolute terms confirmed increased $^{99m}$Tc-MIBI efflux with increased MRP expression. This indicates that $^{99m}$Tc-MIBI efflux is not dependent on the absolute cellular levels of $^{99m}$Tc-MIBI, but efflux is dependent on the rate of transport per minute. Co-incubation of $^{99m}$Tc-MIBI with vincristine resulted in higher $^{99m}$Tc-MIBI levels in all $GLC_4$ cell lines. The fact that $^{99m}$Tc-MIBI also increased by vincristine co-incubation in the parental $GLC_4$ cell line can be explained by a low MRP expression in this cell line. The effects of vincristine are most likely due to partial blocking of $^{99m}$Tc-MIBI efflux by vincristine.

Doxorubicin resistance in $GLC_4/ADR_{150x}$ is multifactorial and considered to be due to an increase in MRP, an increased detoxification and decreased DNA topoisomerase II level (Zijlstra et al, 1987; Timmer-Bosscha et al, 1989; Simon et al, 1994). Therefore, we also analysed the effect of $^{99m}$Tc-MIBI on the non-small-cell lung carcinoma cell line S1 and its *MRP*-transfected subline S1-MRP. Just as in the $GLC_4$ cell lines, a lower accumulation and increased efflux of $^{99m}$Tc-MIBI was observed in S1-MRP compared with the parental S1 cell line.

Recently, it was shown that MRP functions as a glutathione *S*-conjugate carrier (Jedlitschky et al, 1994; Leier et al, 1994; Müller et al, 1994). This finding has stimulated studies with MDR drugs in MRP-overexpressing cell lines after glutathione depletion with BSO (Meijer et al, 1991; Versantvoort et al, 1995*b*; Zaman et al, 1995). Zaman et al (1995) observed a complete reversal of resistance to doxorubicin, daunorubicin, vincristine and etoposide after glutathione depletion in the *MRP*-transfected cell line S1-MRP. Glutathione depletion also resulted in an increased $^{99m}$Tc-MIBI accumulation from *MRP*-transfected cells. These BSO effects were not observed in P-gp-overexpressing cell lines (Versantvoort et al, 1995*b*). In the present study, it was shown that glutathione depletion in $GLC_4/ADR_{150x}$ almost fully restored the cellular $^{99m}$Tc-MIBI level to the level obtained in $GLC_4$. In addition, evaluation of the enhancing effects of $^{99}$Tc-MIBI on vincristine cytotoxicity with isobologram analysis according to Steel and Peckman (1979) demonstrated that the blocking effects of $^{99}$Tc-MIBI on vincristine cytotoxicity in $GLC_4/ADR_{150x}$ is synergistic. Cytotoxicity testing revealed cross-resistance for $^{99}$Tc-MIBI between $GLC_4/ADR_{150x}$ and $GLC_4$. The increased $^{99m}$Tc-MIBI efflux, the effects of glutathione depletion on cellular $^{99m}$Tc-MIBI levels and the cross-resistance for $^{99}$Tc-MIBI as well as increased cytotoxicity of vincristine induced by $^{99}$Tc-MIBI in MRP-overexpressing cells indicate that Tc-MIBI is a substrate for MRP. Tc-MIBI seems to behave in a way similar to that of the chemotherapeutic drugs involved in MRP-mediated MDR. Glutathione *S*-conjugates are transported by MRP (Ishikawa, 1992) and, based on experiments with inside-out vesicles, it is suggested that only substrates with a hydrophobic part and at least two negative charges can be transported by MRP. There is still much debate in the literature on how doxorubicin and vincristine are transported by MRP (Versantvoort et al, 1995*b*; Zaman et al, 1995). For these chemotherapeutic drugs, no glutathione *S*-conjugates have been identified. One possibility might be that these conjugates are unstable and therefore not detectable. Another hypothesis is that the chemotherapeutic drugs are co-transported with glutathione by the MRP pump (Jedlitschky et al, 1994; Leier et al, 1994; Müller et al, 1994; Zaman et al, 1995).

$^{99m}$Tc-MIBI is a synthetic $\gamma$-ray-emitting organotechnetium complex. In vitro, it is a substrate for both P-gp and MRP. Therefore, it might be used in vivo for functional efflux imaging in tumours. Efflux of $^{99m}$Tc-MIBI could then be extrapolated to the efflux of chemotherapeutic agents. This is of potential interest as it is known that in drug-resistant cells both P-gp and MRP can be overexpressed at the same time (Brock et al, 1995; Schuurhuis et al, 1995). For P-gp, several blockers are known, such as verapamil and cyclosporin A, which enables the study of the functional efflux inhibition of $^{99m}$Tc-MIBI by these blockers from P-gp-positive tumours. We are aware of the fact that verapamil and cyclosporin A are not specific inhibitors for P-gp and affect to some extent MRP (Twentyman et al, 1996). For MRP, it is suggested that probenecid and sulfinpyrazone may be useful as specific reversal agents for MRP-mediated drug resistance (Evers et al, 1996). Therefore, clinical studies focusing on inhibition of $^{99m}$Tc-MIBI efflux with such compounds from MRP-positive tumours could be a topic of further investigation.

### ABBREVIATIONS

ATP, adenosine triphosphate; BSO, D,L-buthionine S,R-sulphoximine; DMEM, Dulbecco's modified Eagle medium; DMF, dose-modifying factor; FCS, fetal calf serum; $IC_{10}$, drug concentration inhibiting survival by 10%; $IC_{25}$, drug concentration inhibiting survival by 25%; MDR, multidrug resistance; MRP, multidrug resistance-associated protein; MTA, microculture tetrazolium assay; MTT, 3-(4,5-dimethylthiazol-2-yl)-2,5-diphenyl tetrazolium bromide; P-gp, P-glycoprotein; PBS, phosphate-buffered saline (0.14 M sodium chloride, 2.7 mM potassium chloride, 6.4 mM disodium hydrogen phosphate, 1.5 mM potassium dihydrogen phosphate, pH 7.4); RPMI 1640, Roswell Park Memorial Institute 1640; Tc, Technetium; $^{99m}$Tc-MIBI, $^{99m}$Tc-sestamibi; $^{99}$Tc-MIBI, $^{99}$Tc-sestamibi.

### ACKNOWLEDGEMENTS

The authors wish to thank MC Jeronimus-Stratingh, Department of Pharmacy, for performing mass spectrometry experiments. This study was supported by grant GUKC 94-783 of the Dutch Cancer Society.

### REFERENCES

Ballinger JR, Hua HA, Berry BW, Firby P and Boxen I (1995) $^{99m}$Tc-sestamibi as an agent for imaging P-glycoprotein-mediated multidrug resistance: in vitro and in vivo studies in a rat breast tumour cell line and its doxorubicin-resistant variant. *Nucl Med Comm* **16**: 253–257

Bradley G, Juranka PF and Ling V (1988) Mechanism of multidrug resistance. *Biochem Biophys Acta* **948**: 87–128

Brock I, Hipfner DR, Nielsen BS, Jensen PB, Deeley RG, Cole SPC and Sehested M (1995) Sequential coexpression of the multidrug resistance genes MRP and mdr1 and their products in VP-16 (etoposide)-selected H69 small cell lung cancer cells. *Cancer Res* **55**: 459–462

Cole SPC, Bhardwaj G, Gerlach JH, Mackie JE, Grant CE, Almquist KC, Stewart AJ, Kurz EU, Duncan MV and Deeley RG (1992) Overexpression of a transporter gene in a multidrug-resistant human lung cancer cell line. *Science* **258**: 1650–1654

De Jong S, Zijlstra JG, De Vries EGE and Mulder NH (1990) Reduced DNA topoisomerase II activity and drug-induced DNA cleavage activity in an adriamycin-resistant human small cell lung carcinoma cell line. *Cancer Res* **50**: 304–309

De Vries EGE, Meijer C, Timmer-Bosscha H, Berendsen HH, De Leij L, Scheper RJ and Mulder NH (1989) Resistance mechanisms in three human small cell lung cancer cell lines established from one patient during clinical follow up. *Cancer Res* **49**: 4175–4178

© Cancer Research Campaign 1998

Endicott JA and Ling V (1989) The biochemistry of P-glycoprotein-mediated multidrug resistance. *Ann Rev Biochem* **58**: 137–171

Evers R, Zaman GJR, Van Deemter L, Jansen H, Calafat J, Oomen LCJM, Oude Elferink RPJ, Borst P and Schinkel AH (1996) Basolateral localization and export activity of the human multidrug resistance-associated protein (MRP) in polarized pig kidney cells. *J Clin Invest* **97**: 1211–1218

Ishikawa T (1992) The ATP-dependent glutathione S-conjugate export pump. *Trends Biochem Sci* **17**: 463–468

Jedlitschky G, Leier I, Bucholz U, Center M and Keppler D (1994) ATP-dependent transport of glutathione S-conjugates by the multidrug resistance-associated protein. *Cancer Res* **54**: 4833–4836

Leier I, Jedlitschky G, Bucholz U, Cole SPC, Deeley RG and Keppler D (1994) The MRP gene encodes an ATP-dependent export pump for leukotriene c-4, and structurally related conjugates. *J Biol Chem* **269**: 27807–27810

Meijer C, Mulder NH and De Vries EGE (1990) The role of detoxification systems in resistance of tumor cells to cisplatin and adriamycin. A review. *Cancer Treatm Rev* **17**: 389–407

Meijer C, Mulder NH, Timmer-Bosscha H, Peters WHM and De Vries EGE (1991) Combined in-vitro modulation of adriamycin resistance. *Int J Cancer* **49**: 582–586

Müller M, Meijer C, Zaman GJR, Borst P, Scheper RJ, Mulder NH, De Vries EGE and Jansen PLM (1994) Overexpression of the multidrug resistance associated protein (MRP) gene results in increased ATP-dependent glutathione S-conjugate transport. *Proc Natl Acad Sci USA* **91**: 13033–13037

Pastan I and Gottesman MM (1987) Multiple-drug resistance in human cancer. *N Engl J Med* **316**: 1388–1393

Piwnica-Worms D, Chiu ML, Budding M, Kronauge JF, Kramer RA and Croop JM (1993) Functional imaging of multidrug resistance P-glycoprotein with an organotechnetium complex. *Cancer Res* **53**: 977–984

Scheper RJ, Broxterman HJ, Scheffer GL, Kaaijk P, Dalton WS, Van Heijningen THM, Van Kalken C, Slovak ML, De Vries EGE, Van der Valk P, Meijer CJLM and Pinedo HM (1993) Overexpression of $M_r$ 110,000 vesicular protein in non-P-glycoprotein-mediated multidrug resistance. *Cancer Res* **53**: 1475–1479

Schuurhuis GJ, Broxterman HJ, Ossenkoppele GJ, Baak JPA, Eekman CA, Kuiper CM, Feller N, Van Heijningen THM, Klumper E, Pieters R, Lankelma J and Pinedo HM (1995) Functional multidrug resistance phenotype associated with combined overexpression of Pgp/mdr1 and MRP together with 1-β-D-arabinofuranosylcytosine sensitivity may predict clinical response in acute myeloid leukemia. *Clin Cancer Res* **1**: 81–93

Simon SM and Schindler M (1994) Cell biological mechanisms of multidrug resistance in tumor. *Proc Natl Acad Sci USA* **91**: 3497–3504

Steel GG and Peckman MJ (1979) Exploitable mechanisms in combined radiotherapy: the concept of additivity. *Int J Radiat Oncol Biol Phys* **5**: 85–91

Sugawara I, Kataoka I, Morishita T, Hamada H, Tsuruo T, Itoyama S and Mori S (1988) Tissue distribution of P-glycoprotein encoded by a multidrug-resistant gene as revealed by a monoclonal antibody, MRK16. *Cancer Res* **48**: 1926–1929

Thiebaut F, Tsuruo T, Hamada H, Gottesman MM, Pastan I and Wilingham MC (1987) Cellular localization of the multidrug resistance gene product in normal human tissues. *Proc Natl Acad Sci USA* **84**: 7735–7738

Timmer-Bosscha H, Hospers GAP, Meijer C, Mulder NH, Muskiet FAJ, Martini DRA and De Vries EGE (1989) Influence of docosahexaenoic acid on cisplatin resistance in a human small cell lung carcinoma cell line. *J Natl Cancer Inst* **81**: 1069–1075

Twentyman PR and Versantvoort CHM (1996) Experimental modulation of MRP (multidrug resistance-associated protein)-mediated resistance. *Eur J Cancer* **32A**: 1002–1009

Vallabhaneni VR, Chiu ML, Kronauge JF and Piwnica-Worms D (1994) Expression of recombinant human multidrug resistance P-glycoprotein in insect cells confers decreased accumulation of technetium-99m-sestamibi. *J Nucl Med* **35**: 510–515

Versantvoort CHM, Broxterman HJ, Pinedo HM, De Vries EGE, Feller N, Kuiper CM and Lankelma J (1992) Energy-dependent processes involved in reduced drug accumulation in multidrug-resistant human lung cancer cell lines without P-glycoprotein expression. *Cancer Res* **52**: 17–23

Versantvoort CHM, Withoff S, Broxterman HJ, Kuiper CM, Scheper RJ, Mulder NH and De Vries EGE (1995*a*) Resistance associated factors in human small cell lung carcinoma $GLC_4$ sublines with increasing adriamycin resistance. *Int J Cancer* **61**: 375–380

Versantvoort CHM, Broxterman HJ, Bagrij T, Scheper RJ and Twentyman PR (1995*b*) Regulation by glutathione of drug transport in multidrug-resistant human lung tumor cell lines overexpressing multidrug resistance-associated protein. *Br J Cancer* **72**: 82–89

Wishart GC, Bissett D, Paul J, Jodrell D, Harnett A, Habeshaw T, Kerr DJ, Macham MA, Soukop M, Leonard RCF, Knepil J and Kaye SB (1994). Quinidine as a resistance modulator of epirubicin in advanced breast cancer: mature results of a placebo-controlled randomized trial. *J Clin Oncol* **12**: 1771–1777

Zaman GJR, Flens MJ, Van Leusden MR, De Haas M, Mülder HS, Lankelma J, Pinedo HM, Scheper RJ, Baas F, Broxterman HJ and Borst P (1994) The human multidrug resistance-associated protein (MRP) is a plasma membrane drug-efflux pump. *Proc Natl Acad Sci USA* **91**: 8822–8826

Zaman GJR, Lankelma J, Van Tellingen O, Beijnen J, Dekker H, Paulusma C, Oude Elferink RPJ, Baas F and Borst P (1995) Role of glutathione in the export of compounds from cells by the multidrug-resistance-associated protein. *Proc Natl Acad Sci USA* **92**: 7690–7694

Zijlstra JG, De Vries EGE and Mulder NH (1987) Multifactorial drug resistance in an adriamycin-resistant human small cell lung carcinoma cell line. *Cancer Res* **47**: 1780–1784

© Cancer Research Campaign 1998