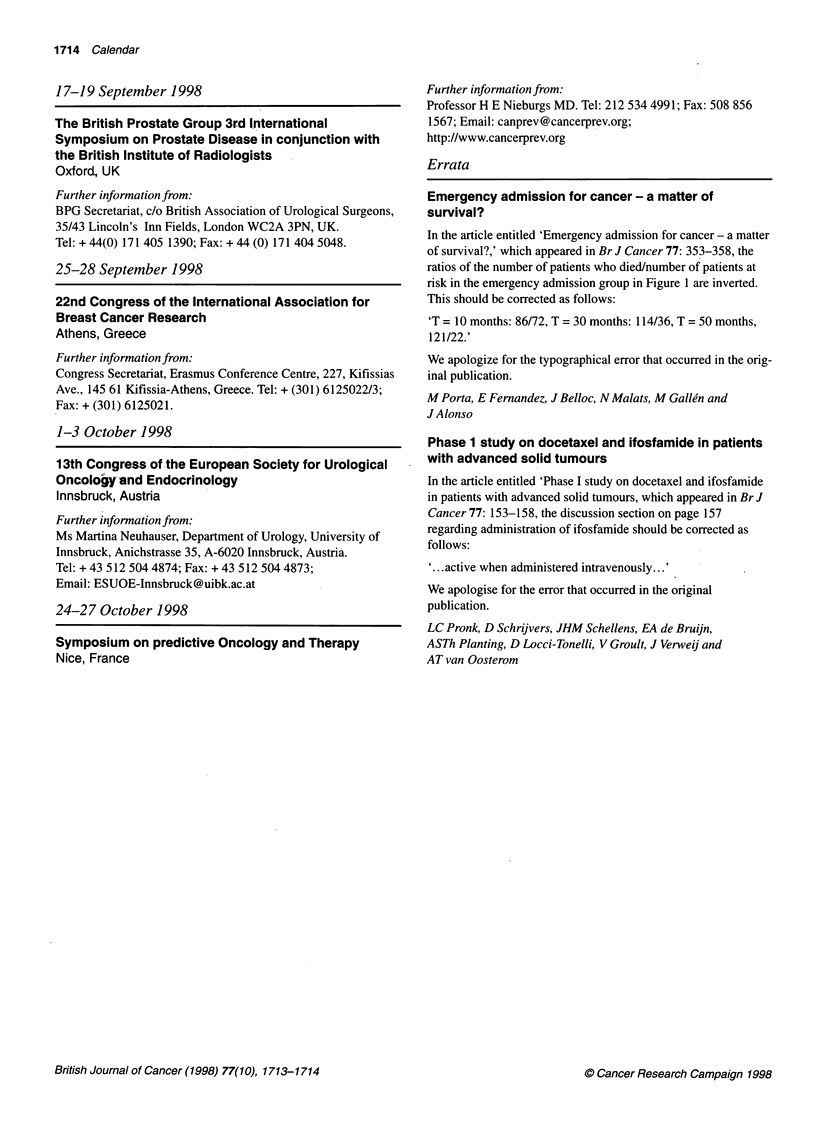# Emergency admission for cancer - a matter of survival?

**Published:** 1998-05

**Authors:** 


### *Errata*

### Emergency admission for cancer – a matter of survival?

In the article entitled 'Emergency admission for cancer – a matter of survival?,' which appeared in *Br J Cancer* **77**: 353–358, the ratios of the number of patients who died/number of patients at risk in the emergency admission group in Figure 1 are inverted. This should be corrected as follows:

'T = 10 months: 86/72, T = 30 months: 114/36, T = 50 months, 121/22.'

We apologize for the typographical error that occurred in the original publication.

*M Porta, E Fernandez, J Belloc, N Malats, M Gallén and J Alonso*